# Intracellular HIV-1 Gag localization is impaired by mutations in the nucleocapsid zinc fingers

**DOI:** 10.1186/1742-4690-4-54

**Published:** 2007-08-03

**Authors:** Boyan Grigorov, Didier Décimo, Fatima Smagulova, Christine Péchoux, Marylène Mougel, Delphine Muriaux, Jean-Luc Darlix

**Affiliations:** 1 LaboRetro, Unité de virologie humaine INSERM U758, IFR128, ENS, 46 allée d'Italie, 69 364 Lyon, France; 2 CPBS, UMI, CNRS, 4 bd Henri IV, 34000 Montpellier, France

## Abstract

**Background:**

The HIV-1 nucleocapsid protein (NC) is formed of two CCHC zinc fingers flanked by highly basic regions. HIV-1 NC plays key roles in virus structure and replication *via *its nucleic acid binding and chaperoning properties. In fact, NC controls proviral DNA synthesis by reverse transcriptase (RT), gRNA dimerization and packaging, and virion assembly.

**Results:**

We previously reported a role for the first NC zinc finger in virion structure and replication [1]. To investigate the role of both NC zinc fingers in intracellular Gag trafficking, and in virion assembly, we generated series of NC zinc fingers mutations. Results show that all Zinc finger mutations have a negative impact on virion biogenesis and maturation and rendered defective the mutant viruses. The NC zinc finger mutations caused an intracellular accumulation of Gag, which was found either diffuse in the cytoplasm or at the plasma membrane but not associated with endosomal membranes as for wild type Gag. Evidences are also provided showing that the intracellular interactions between NC-mutated Gag and the gRNA were impaired.

**Conclusion:**

These results show that Gag oligomerization mediated by gRNA-NC interactions is required for correct Gag trafficking, and assembly in HIV-1 producing cells and the release of infectious viruses.

## Background

The retroviral Gag polyprotein precursor is formed of three essential domains, namely the matrix (MA), the capsid (CA) and the nucleocapsid (NC), which upon protease mediated processing of Gag constitute the architecture of the infectious mature viral particle. The three Gag domains contain the critical determinants that orchestrate virus assembly in the infected cell, via membrane-MA, CA-CA and NC-gRNA interactions [2-8]. In the mature virus, the MA protein is located under the virion envelope, which derives from the infected cell membrane. In the case of HIV-1, MA is myristoylated and contains basic amino acids within its N-terminus required for Gag-membrane binding and determinants that specifically interact with the cellular adaptator proteins AP-3 and AP-2. These AP proteins contribute to the intracellular transport of Gag to endosomal compartments and retroviral budding [9-11]. The CA molecules form the outer shell of the viral core while NC molecules extensively coat and condense the gRNA in the interior of the virion core [2]. HIV-1 NC contains two zinc fingers flanked by basic regions and is located at the C-terminus of Gag, followed by the p6 domain. This later p6 domain is required for particle budding during which the viral particles pinch-off from the cellular membrane (reviewed in [5]. The p6 domain contains a Proline-rich and a di-Leucine domains, which are the target of the cellular proteins Tsg101 and Alix, respectively, involved in the cellular class E protein sorting pathway and the HIV-1 budding machinery [5,12,13].

HIV-1 NC has been extensively studied during the past 15 years and was shown to be implicated in virus structure, gRNA dimerization and proviral DNA synthesis [3,4,7]. The highly basic nature of NC makes it a partner of choice of RNA while the zinc fingers appear to provide specific recognition of the HIV-1 Psi packaging signal necessary for gRNA packaging [14]. Furthermore, specific RNA-NC interactions promote Gag-Gag oligomerization which turns out to be a prerequisite for assembly and virus biogenesis [15-18]. Both NC zinc fingers and basic domains are essential for virus formation and infectivity [1,16,17,19-21]. Mutations in NC basic residues cause defects in Gag-viral RNA interactions and thus in HIV-1 assembly and budding [15,16,22]. More recently, new insights into the role of NC in Gag assembly show that mutations and deletions in the basic residues of NC prevent Gag-Gag multimerization but not Gag association with cellular membranes [23].

In the present study, we explored the influence of the NC zinc fingers in HIV-1 assembly by analyzing intracellular Gag and gRNA localization, Gag/membrane association and virion morphogenesis.

## Methods

### Plasmid DNA

HIV-1 pNL4-3 DNA was provided by the National Institute of Health, USA. The HIV-1 ΔZF1 and H23C Gag mutant DNA constructs were described elsewhere [1]. The HIV-1 GagΔNC proviral DNA construct [24] was provided by A.Cimarelli. The HIV-1 ΔZF2 and H44C Gag mutants were obtained by site directed mutagenesis on the pNL4.3 HIV-1 molecular clone as described [1] using the following oligonucleotides 5'CCTGTCTCTCAGTACCGCCCTTTTTCCTAG3' and 5'CTTTCATTTGGCATCCTTCC3', respectively. The double ΔZF1ZF2 and H23C/H44C Gag mutants were obtained by cloning the ApaI-AgeI fragments of H44C and ΔZF2 into the H23C and ΔZF1 pNL4.3 mutant clone, respectively. The pcDNA3.1 plasmid (Clonetech) was used as a control DNA vector.

### Mammalian cell culture, DNA transfection and virus production

The human 293T cell line, HeLa P4 cells expressing the CD4 receptor and the LacZ gene under the control of the HIV-1 LTR and HeLa cells used were grown in Dulbecco's modified essential medium (DMEM), all supplemented with 10% fetal calf serum and antibiotics. 293T were transfected using the calcium phosphate method [18]. For immunofluorescence staining, HeLa cells were transfected with DNA using the Fugene^® ^transfection method (Invitrogen). To analyse virus production, cells were washed with PBS and medium was changed 5 h post-transfection. Culture supernatants containing virus particles were harvested 24 hours later and clarified by filtration (0.45 μm, Nalgen). The cells were then washed and lysed with 0,5% Triton-PBS.

### Virus preparation

Virions were purified from filtered culture supernatants by pelleting them through a cushion of 20% sucrose in TNE (100 mM NaCl, 10 mM Tris HCl, pH 7.4 and 1 mM EDTA) at 35 K rpm for 1 h in a Beckman SW41 rotor.

### CAp24 antigen ELISA

To measure viral production, a CAp24 ELISA test was used. Aliquots of the same volume of viral supernatants (free CAp24 + virion associated = S) and pellet virions by ultracentrifugation (V) were resuspended in cell media with 0.5% Triton, and administered on 96 well plates coated with 10 μg/ml anti-CAp24 antibodies (23A5G and 3D10G9B8, BioMérieux) and then blocked with 10% horse serum in PBS-0.05% Tween-20. A biotinylated anti-CAp24 antibody (bioMérieux) was added and the ELISA was revealed with streptavidin and orthophenylene-diamine (OPD)-H_2_O_2 _(Sigma). The plate was read on ELISA-reader at 490 and 630 nm.

### HIV Infectivity assays

Virus infectivity was assessed on HeLaP4 cells as described in [25]. The infectivity was determined by counting the number of blue cells.

### Genomic RNA analysis by Dot-Blot

For viral RNA analysis, virus pellets were resuspended in TNE buffer and lysed in 1% SDS, 100 μg of proteinase K per ml. Nucleic acids were extracted twice with phenol-chloroform and ethanol precipitated. Pellets were resuspended in DNase buffer (40 mM Tris-HCl, pH 7.5, 6 mM MgCl2, 10 mM NaCl, 10 mM dithiotreitol, 200 U of RNasin per ml) and contaminant plasmid DNA was digested with RQ1 DNase (100 U/ml) for 20 min at 37°C. RNA was purified by phenol-chloroform extraction, ethanol precipitated and resuspended in water. Hybridization with a random 32P-labeled 5.3 kb SacI-SalI fragment of the pNL4-3 plasmid corresponding to gag and pol sequences and quantitative analyses were done as previously described [19].

### Subcellular fractionation

Twenty-four hours post transfection, 293T cells were washed with PBS and removed from the plate in PBS-1 mM EDTA, pelleted by centrifugation at 600 × g, resuspended in 1 ml of a homogenization buffer containing 10 mM Tris-HCl, pH 7.5, 0.25 M sucrose, 1 mM EDTA and protease inhibitors (Complete Mini EDTA-free from Roche), and then fragmented using a glass homogenizer. Nuclei were eliminated by centrifugation at 600 × g for 10 min at 4°C. The resulting post-nuclear supernatant (PNS) was subjected to subcellular fractionation on OptiPrep^® ^gradient for the separation of different membrane compartments as described elsewhere [25]. Fractions were collected and proteins were analyzed by SDS-PAGE and immunoblotting.

### Immunoblotting

Viral proteins were separated on 10% SDS-PAGE and detected by immunoblotting with a mouse anti-CAp24 (P3D10G9B8, BioMérieux), and the cellular protein in the gradient was detected with the mouse anti-Lamp2 (Santa Cruz Biotechnology Inc.). The corresponding immunoglobulins conjugated with horse radish peroxidase (HRP) (DakoCytomation) were used and the signal was detected using SuperSignal^® ^West Pico Chemiluminescent Substrate (Pierce).

### RT-PCR

Fractions from Optiprep^® ^gradients were resuspended in equal volumes of a lysis buffer containing 100 mM Tris-HCl, pH 7.4, 20 mM EDTA, 2% SDS, 200 mM NaCl and 200 μg/ml proteinase K and incubated at 37°C for 30 min. RNA was purified by phenol/chloroform extraction, and precipitated with ethanol. RNA samples were pelleted by centrifugation at 4°C, 14 000 rpm for 30 min, and resuspended in RNAse-free water. Contaminant DNA was eliminated by digestion with RQ1 DNAse. RNA aliquots were reverse transcribed using the Invitrogene RT assay. The RT reaction was followed by PCR of the cDNA using primers for the cPPT as follows: up cPPT- nt. 4775 GCGCGATCGATCCACAATTTTAAAAGAAAAGGGGGGATTG, and down cPPT- nt. 4907 GCGCGATCGATTGTAATAAACCCGAAAATTTTG. The PCR DNA product of 132 bp was separated on a 2% agarose gel and visualized by ethidium bromide staining. The gel images were quantified by Metamorph software and semi-quantitave analysis of the gRNA was evaluated.

### Immunofluorescence staining and confocal microscopy imaging

293T cells grown on poly-lysine coated coverslips and HeLa cells were transfected and, 24 h later, fixed in 3% paraformaldehyde-PBS for 20 min. After fixation, cells were permeabilized using 0.2 % Triton, and then incubated in 1% BSA-PBS with primary antibodies: mouse anti-CAp24 (BioMérieux or NIH), rabbit anti-MAp17 (NIH, USA), mouse anti-Lamp1 and anti-Lamp3 (Santa Cruz Biotechnology Inc.). The corresponding fluorescent Alexa^® ^488 and 546-conjugated secondary antibodies were used (Molecular probes). Coverslips were washed and mounted on microscope slides with Mowiol (Sigma). Images were acquired on Axioplan 2 Zeiss CLSM 510 confocal microscope with Argon 488/458, HeNe 543 lasers and plan apochromat 63× 1.4 oil objective, supplied with LSM 510 software. The percentage of colocalization (merge signals) was evaluated by the Metamorph software (UIC).

### Fluorescent in situ hybridization (FISH)

Transfected 293T or HeLa cells were grown on poly-lysine treated coverslips and 24 h post-transfection were washed with PBS and fixed as described before [26] and stored at 4°C in 70% ethanol. Detection of the HIV-1 genomic RNA was performed by FISH [26] with a Cy3-conjugated oligonucleotide (GagHIVCy3), corresponding to position 1524 to 1563 of the HIV-1 sequence (MWG-Biotech). The probe was adjusted to 1 ng/ul in 66% formamide, 0.2× SSC, 2 μg/μl tRNA and 2 μg/μl of sheared salmon sperm DNA. After a denaturation step of 5 min at 95°c, the probe was mixed V/V with a solution containing 20% sulfate dextran, 4 × SSC, 0,04% RNase-free BSA and 4 mM Vanadyl-ribonucleoside complex, and applied to each coverslip. Hybridization was performed at 37°C overnight in a humid chamber. 24 hs later, the coverslips were washed twice with 50% formamide, 2× SSC at 37°c, followed by 3 washes with 50% formamide, 1× SSC at 37°c, and mounted on a slide in Vectashield with DAPI (Vector Laboratories Inc.). Image acquisition and analysis were performed in the Montpellier RIO Imaging microscopy facility. Images were taken with a Leica DMRA wide-field microscope and acquisition was performed with a Coolsnap HQ camera driven by Metamorph software.

### Transmission electron microscopy

HeLaP4 cells expressing HIV-1 or either one of the NC zinc finger mutants were fixed in 2% glutaraldehyde, 0.1 M Sörensen phosphate buffer, pH 7.4 for 30 min at 4°C. Then, cells were washed 3 × 10 min with phosphate buffer containing 0.2 M sucrose and post-fixed in 1% OsO4 that was 1.5% with respect to potassium ferrocyanide for 1 hr at room temperature. Cells were dehydrated through graded ethanol and embedded in Epon 812. Thin sections were cut and picked up on 200 mesh copper grids, stained with uranyl acetate and counter-stained with lead citrate. Specimens were analyzed with a Philips CM120 electron microscope (CMEABG – Villeurbanne – France).

## Results

### 1- Mutations in NC zinc fingers impair virus production, maturation, gRNA packaging and infectivity

**Figure 1:**
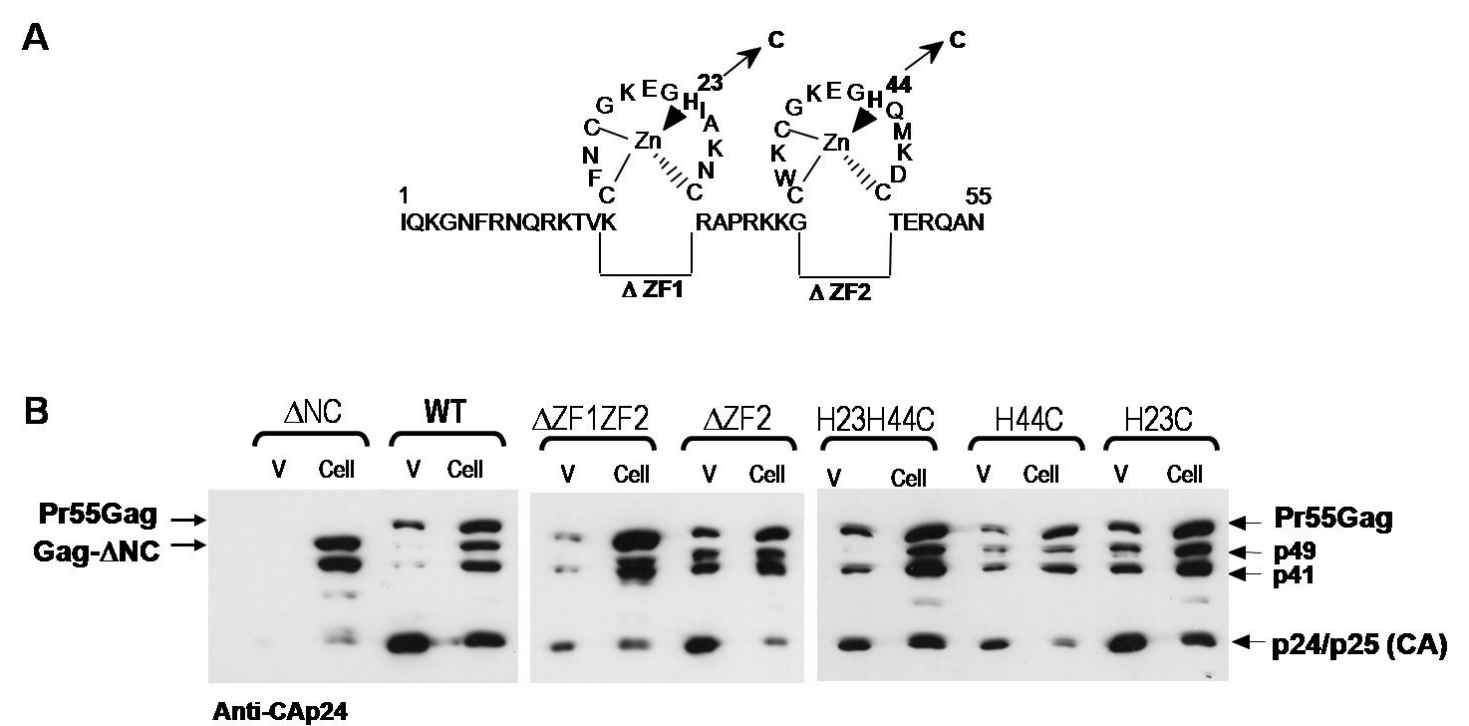
**A. The HIV-1 NC Zinc finger mutants**. The sequence of HIV-1 NCp7 (1–55) is shown. Mutations H23C and H44C are indicated. Deletions ΔZF1 and ΔZF2 correspond to a complete deletion of the zinc fingers (ZF). ΔNC was described elsewhere [24]. **B. Gag expression and maturation in HeLaP4 cells**. HelaP4 cells were transfected with the pNL4.3 DNA (wild-type or either one of the NC mutants) and subsequently harvested and lysed. Viral proteins were analyzed by SDS-PAGE and revealed by immunoblotting with anti-CAp24. Immunodetection of the Gag maturation products. Wild-type HIV-1 and NC mutants are indicated. Lanes "V" and "Cell" representing pelletable virions from culture medium and cell lysates, respectively. Pr55Gag, p41(MA-CA), p49(MA-CA-p2-NC) and CAp24/p25 are indicated by arrows.

Mutations were generated in the HIV-1 NC zinc fingers (ZF), namely ZF1 and ZF2, so as to change or impair Zn^2+ ^coordination known to modify the central globular domain of NC formed by the ZFs [1,19,21,27,28]. Four HIV-1 constructs containing ZF mutations were analyzed, namely H44C, H23H44C, ΔZF2 and ΔZF1ZF2 (Fig. 1A), while two additional mutants, namely H23C and ΔZF1 have previously been characterized [1]. HIV-1 wild-type and ZF mutant virions (V) and cell lysate (Cell) were recovered and analyzed by immunoblotting (Fig. 1B).

To monitor the impact of the ZF mutations on virus production, the levels of CAp24 present in the supernatant (S) or as viral particles (V) were determined by ELISA (Table 1). In comparison with wild-type, all the ZF mutants were impaired for CAp24 release, reducing particle production by more than two fold, with a 3 to 5-fold reduction for the mutations affecting both zinc fingers in comparison with wild-type (Table 1). Furthermore, when the amounts of virus (V) were determined and compare to the total CAp24 production, the impact of the ZF mutations was found to be even more pronounced since deleting or mutating the second ZF extensively decreased particle production found to be below 10% of the wild-type level (Table 1, column V). The most drastic effect was observed when both ZFs were deleted because less than 1% of virus was released in particular for ΔZF1ZF2 mutant, just as for an HIV-1 mutant carrying the complete deletion of NC (Table 1, column V). Very similar results were obtained for the HIV-1 NC-ZF mutants expressed in 293T cells (data not shown). In conclusion, in both 293T and HeLaP4 cells mutating or deleting both ZFs prevented the proper assembly and efficient production of HIV-1.

**Table 1:** Properties of HIV-1 NC zinc fingers mutants produced by HeLa-P4 cells

Virus	total p24 release (S)	% of p24 in virions (V)	relative levels of gRNA in virions	Infectivity
wt	100%	100%	100%	+
H23C	37 ± 9	31.5 ± 4.5	10 ± 3	-^b^
H44C	18.5 ± 9.5	3 ± 1	3.5 ± 1.5	-
ΔZF2	43 ± 6	8.3 ± 0.5	5 ± 1	-
H23CH44C	24.7 ± 12	17 ± 7	4 ± 0	-
ΔZF1ZF2	23.7 ± 10	1 ± 0.1	3.5 ± 0.5	-
ΔNC	~10	0.5 ± 0.1	nd	-^a^

Viral production was assessed by Elisa test: The percentage of CAp24 found in filtered viral supernatant and in virions after ultracentrifugation (V) in comparison to wt (the numbers are representative of at least 2 experiments). The relative level of gRNA in virions was assessed by dot blot. The infectivity was assessed on HeLaP4 relative to the same amount of gRNA. References: (a) by [24]; (b) by [1]. Note that ΔZF1 is described in [1].

Viral proteins present in cells and viral particles were analyzed by immunoblotting using anti-CAp24 (Fig. 1B). In wild-type virions, the vast majority of Gag has been processed (Fig. 1B, lane wt, V) while large amounts of unprocessed Gag and p41/p49 were found in the ZF mutant virions (Fig. 1B, lanes V). In comparison with the wild-type HIV-1, processing of Gag in viral particles was partially changed by mutating the NC zinc fingers, as evidenced by an accumulation of the MA-CA precursor (p41) and probably MA-CA-p2-NC (p49) (Fig. 1B, lanes V). The same defect in Gag processing was observed following mutating or deleting the first ZF [1]. Processing of Gag was modified by the H23H44C mutations and ΔZF1ZF2 deletions, as indicated by the accumulation of partially cleaved Gag products, in particular p41 in virions (Fig. 1B, see arrows; compare lane "V" of these ZF mutants with lane "V" for the WT). In particular, these two NC mutants lack p49; thus the absence of p49 could be due to an undetectable level of protein or that mutations in both ZFs still permit maturation of p49. All the other NC mutants show p41 and p49 accumulation in the virions (Fig. 1B, lanes V), indicating a defect in maturation cleavage between NC-p1 or p1-p6 (that can be due to a conformational change of NC induced by ZF mutation, as previously reported [27]. All NC mutants show intracellular Gag and p41/49 accumulation, except for limited amounts of CAp24/25, suggesting some defect in the budding process (Fig 1B, lanes Cell). These results suggest that ZF mutants have a negative impact on virus budding and Gag processing.

Although the viral production was low, enough virus could be recovered to monitor the relative level of genomic RNA (gRNA) in virions. The level of gRNA in virions was analyzed by dot blot hybridization (not shown) and, as reported in Table 1, none of the NC mutants harbored a wt gRNA level. In fact, mutations in the ZF reduced gRNA levels by 10 to 20 fold in comparison with wild-type HIV-1. Finally, the infectivity of HIV-1 ZF mutants was assessed and none of the mutants were infectious (Table 1).

### 2- Intracellular accumulation and localization of Gag proteins with NC ZF mutations

**Figure 2:**
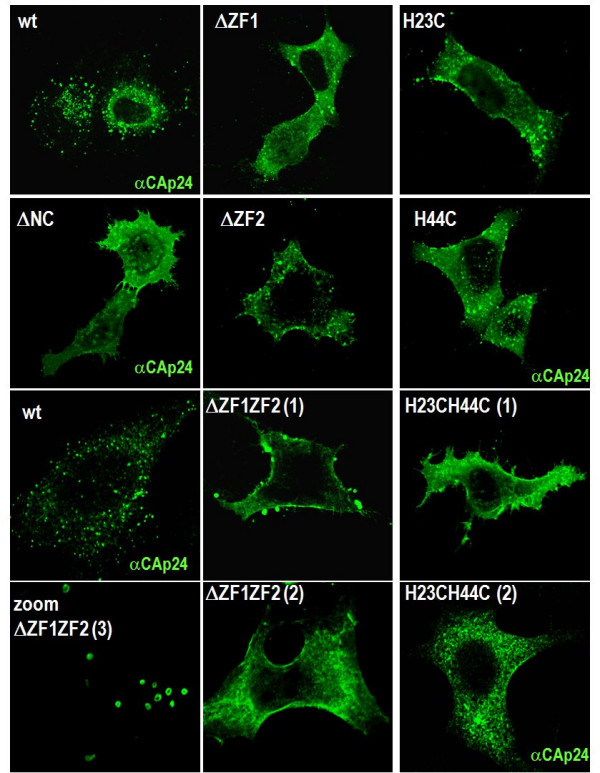
**Localization of HIV-1 Gag carrying NC zinc finger mutations by immunofluorescence microscopy**. Cells were transfected with the indicated viral DNA and then fixed and stained with an anti-CAp24 antibody, as described in material and methods. In addition, cytoplasmic ring-like membranes were found labeled with these two latter Gag mutants (zoomed picture) in less than 1% of the cells. Note that the images obtained for NC(ΔZF1ZF2) was also found for NC(H23H44C).

Since Gag with ZF mutations accumulated in cells, we examined the intracellular Gag localization in HeLa cells by immunofluorescence and confocal microscopy using an anti-CAp24 antibody (Fig. 2). Wild-type HIV-1 Gag displayed a punctuate pattern in the cytoplasm and was found in patches at or near the plasma membrane (PM) (Fig. 2), in agreement with the data of [29,30]. As shown in Fig. 2, NC mutated Gag accumulated at the PM and in the cytoplasm. More precisely, Gag with a mutation or a deletion of the first ZF (ΔZF1 and H23C) preferentially accumulated in patches at the PM while Gag carrying mutations in the second ZF (ΔZF2 and H44C) accumulated at the PM and in intracellular vesicles (Fig. 2). Similar results were observed with 293T cells (data not shown). The most dramatic effect was observed with Gag carrying mutations in both ZFs, namely Gag-ΔZF1ZF2 and Gag-H23H44C, which strongly accumulated at the PM and sometimes in intracellular membranes with "*rings"-like structures *(~1% of the cells) (Fig. 2, ΔZF1ZF2 zoomed picture). In addition, these NC-Gag mutants also showed a very diffuse pattern in the cytoplasm, as if mutated Gag had lost membrane association (Fig. 2, see H23H44C). The complete deletion of NC domain of Gag caused an overall accumulation, where Gag-ΔNC was found essentially at the PM, in the cytoplasm and even sometimes in the nucleus (Fig. 2, ΔNC).

**Figure 3:**
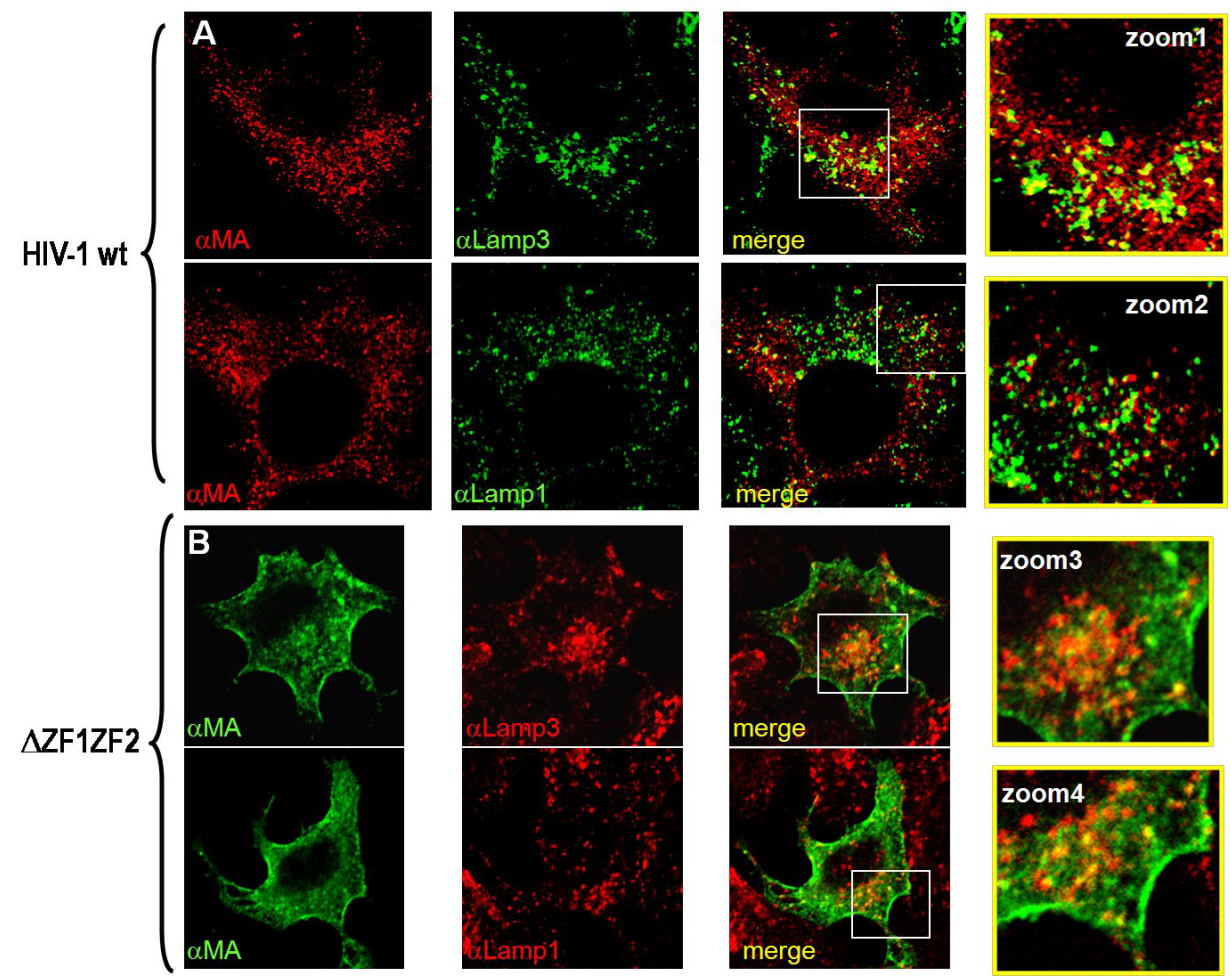
**Plasma membrane accumulation of the HIV-1 NC(ΔZF1ZF2) Gag**. HeLa cells were transfected with wild-type HIV-1 (A) or NC(ΔZF1ZF2) (B) DNA, then fixed and stained for the detection of Gag with an anti-MAp17; with an anti-CD63/Lamp3 for late endosomes, and with an anti-Lamp1 for lysosomes, as indicated. Zoomed-1 picture shows wild-type Gag colocalization with CD63/Lamp3 late endosomal marker (26 ± 6%) and zoomed-2 picture with the Lamp1 marker (6 ± 2%). In contrast, zoomed-3 picture shows less colocalization of this mutant with Lamp3 in comparison to wt (6 ± 4%). Zoomed-4 picture shows an accumulation of NC(ΔZF1ZF2)-Gag mutant at the PM, and less or equal with Lamp1(+) intracytoplasmic vesicles (3 ± 1%).

In order to assess the nature of the membranes where the ZF mutant Gag was found, cells expressing either Gag-ΔZF1ZF2 or Gag-H23H44C were analyzed by immunofluorescence staining using late endosomal (Lamp3) and lysosomal (Lamp1) markers (Fig. 3). Wild-type HIV-1 Gag colocalized with Lamp3-containing vesicles (~30%), i.e. late endosomes, but very few with Lamp1 (less than 10%), i.e. lysosomes (Fig. 3A). For the ΔZF1ZF2 NC mutant, Gag was found either in the cytoplasm, poorly associated with the Lamp1 marker (Fig. 3B), or at the PM. Similar observations were made with the H23H44C NC mutant (data not shown). This latter Gag mutant weakly localized with Lamp3(+) vesicles (7 ± 4%) and with Lamp1(+) vesicles (5 ± 2%). Thus, it appears that the double ZF-mutated Gag accumulated in the cytosol and strongly at the PM, and is delocalized from endosomal membranes in comparison with the wild-type Gag. Taken together these results suggest that upon synthesis NC-mutated Gag molecules are targeted mainly to the PM (or to intracellular ring-shape membranes, that can derived from PM invaginations) where they concentrate, resulting in a strong intracellular Gag retention and a decrease in virus production.

### 3- Influence of ZF mutations on intracellular genomic RNA localization

**Figure 4:**
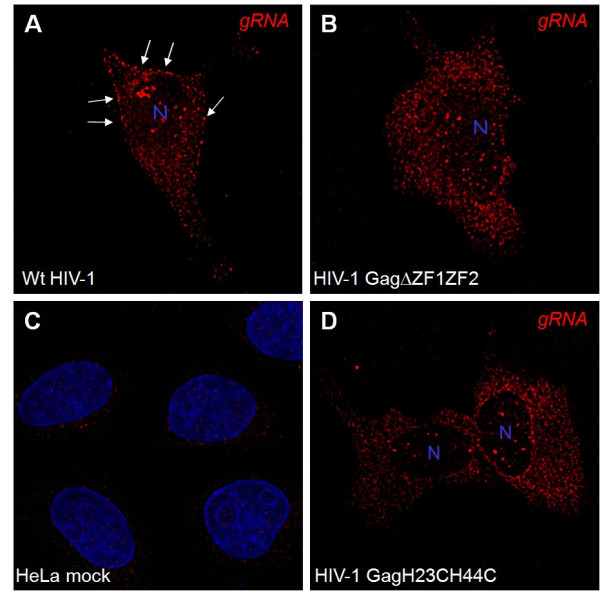
**Intracellular localization of the gRNA in cells expressing the NC zinc finger-mutant Gag**. HeLa cells were transfected with wild-type HIV-1 (A), or NC(ΔZF1ZF2) (B), or NC(H23H44C) (D) DNA, then fixed and stained for the detection of the gRNA by FISH, as described in material and methods. The fluorescent Cy3-labelled oligonucleotide probe hybridized to the HIV-1*gag *gene (in red). The nucleus was stained with Dapi in the "mock" HIV-negative cells (C). The arrows indicate the accumulation of wt gRNA at the PM.

Intracellular localization of the gRNA was assessed by FISH analysis in HeLa cells expressing either HIV-1 wild-type, ΔZF1ZF2- or H23H44C-NC mutant (Fig. 4). For wild-type HIV-1, the gRNA labeling was located in the nucleus, in the cytoplasm and at distinct PM locations (Fig. 4A, see arrows). With the NC mutants, signals were found in the nucleus and in the form of patches in the cytoplasm but not at the PM (Fig. 4B and 4D). Thus, the main difference between the wild-type and the ZF mutants was that the gRNA of the ZF mutants accumulated in the cytoplasm.

These results suggest that ZF-mutated Gag is poorly associated with the gRNA at the cell surface and that the ΔZF1ZF2 and H23H44C NC mutations alter intracellular Gag-gRNA interactions.

### 4- NC ZF mutations prevent intracellular Gag-RNA localization in late endosomes

**Figure 5:**
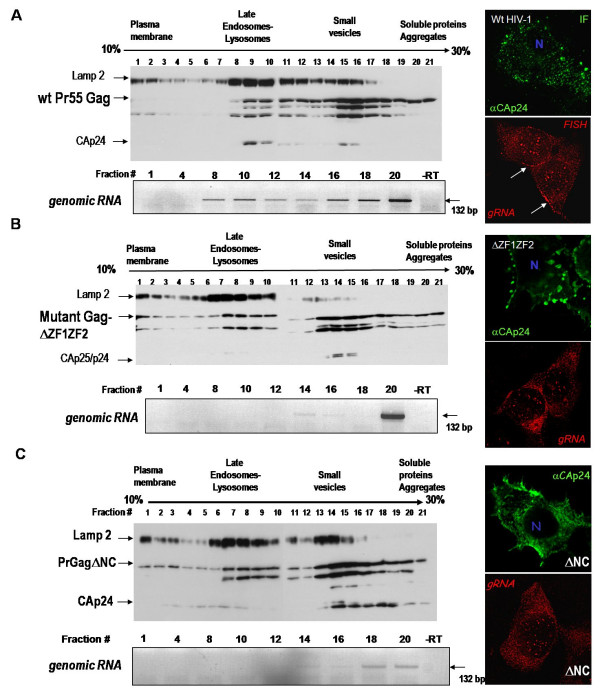
**Subcellular localization of Gag and the gRNA**. Subcellular fractionations of 293T cells expressing wild-type HIV-1 (A, as in (21)) or NC(ΔZF1ZF2) (B) or ΔNC (C) were analyzed by OptiPrep gradient centrifugation. Cells were broken as described in materials and methods and the post-nuclear supernatant (PNS) was fractionated by Optiprep gradient. 20 μl of each fraction were loaded on SDS-PAGE, and Gag and Lamp2 were analyzed by immunoblotting using anti-Cap24 and anti-Lamp2 antibodies. Each fraction of the gradient was tested for the presence of the gRNA by RT-PCR as described in materials and methods. The expected 132 bp DNA fragment was detected on 1% agarose gel. In addition to the gradient analyses, the immunofluorescence (IF) detections are shown, representing the cells stained with an anti-CAp24 (in green) for Gag (A) or mutated Gag (B and C), and the FISH treatment of the 293T cells expressing HIV-1 (A) or the NC Gag mutants (B and C) for the gRNA using the Gag-oligo-Cy3 probe (in red).

The fact that intracellular HIV-1 Gag molecules co-fractionate with late endosomal markers [25] prompted us to examine the localization of ZF-mutated Gag and the gRNA using the same subcellular fractionation and gradient protocols as before (see methods). Post-nuclear supernatants from 293T cells expressing either wild-type HIV-1, the ΔZF1ZF2-NC or ΔNC mutant were fractionated and each fraction was analyzed for its content in viral proteins and gRNA (Fig. 5A, 5B and 5C, respectively). In agreement with our previous findings [25], wild-type Gag was found at the bottom of the gradient together with the gRNA (Fig. 5A, fractions 18–21) and associated with small vesicles or in dense complexes with very few gRNA (fractions 14–16). In the late endosomal/lysosomal fractions, Gag and processed proteins were found together with the gRNA (fractions 8–12), indicating that Gag and the viral RNA are most probably associated in the form of viral ribonucleoprotein complexes. Only small amounts of Gag were present at the PM together with the gRNA (fraction 1). By immunofluorescence microscopy, Gag was present in patches at the PM and in the cytoplasm, ressembling the gRNA pattern by FISH (see IF and FISH pictures, Fig. 5A).

In the case of the ΔZF1ZF2-NC mutant, the subcellular fractionation only reveals the mutated Gag and the viral RNA located at the bottom of the gradient (Fig. 5B, fractions 18–20), probably associated with active ribosomes. Semi-quantitative analysis of the gRNA level by RT-PCR show that 70% of the gRNA for ΔZF1ZF2-NC mutant and 15% for ΔNC-Gag mutant is remaining in comparison with wild-type gRNA (100% in the whole gradient), indicating that deletion of the NC domain results in gRNA instability, possibly due to impaired interactions between Gag and the gRNA. In addition, colocalization of the mutated Gag and the gRNA disappeared at the level of late endosomes and at the PM (Fig. 5B, fractions 7–10, and fraction 1, respectively). Abnormal processed mutated Gag was found in fractions 14–15 in comparison with wild-type Gag, suggesting a defect in Gag targeting and/or budding. As in HeLa cells, we observed by immunofluorescence microscopy of 293T cells that mutated Gag accumulated in endosomal membranes and in discrete domains at the PM (Fig. 5B, see IF). By FISH, the gRNA accumulated in the cytoplasm, and again the PM labeling was lost (Fig. 5B, see FISH). Similar results were obtained upon deletion of NC (Fig. 5C) since GagΔNC was found all over the gradient, in agreement with the immunofluorescence analysis where Gag was evenly distributed within the cell (Fig. 5C, see IF) as well as the gRNA (see FISH). Similar results were obtained with the H23H44C-Gag mutant (data not shown).

Taken together, these results indicate that the ZF mutations impair intracellular Gag/gRNA association, most probably due to an alteration of their interactions.

### 5- Impact of the NC zinc finger mutations on the structure of the viral particles as seen by electron microscopy

**Figure 6:**
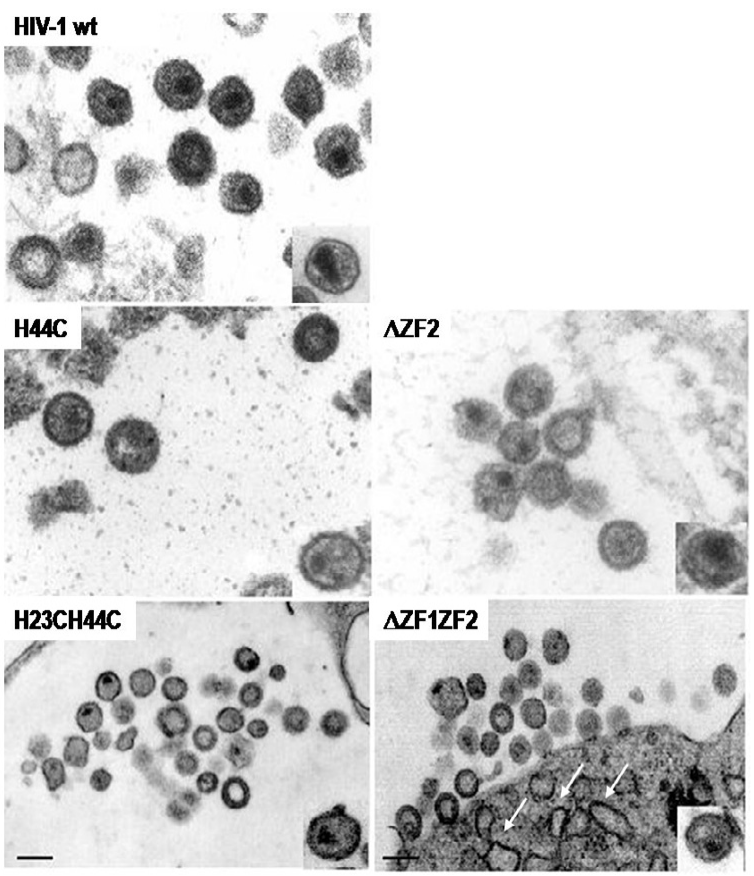
**Electron microscopy analysis of HIV-1 NC mutant virions**. Virions were produced by DNA transfected HeLaP4 cells and further processed as indicated in materials and methods. Bar is 100 nm.

To analyze the influence of ZF-mutations on virus assembly, virions produced by HeLa cells expressing either one of the HIV-1 ZF-mutants were collected and processed for electron microscopy as described before [1] (Fig. 6). Most HIV-1 wild-type virions show a mature morphology with either a conical or central globular nucleocore (Fig. 6, wt). NC mutants H44C and ΔZF2 exhibited either an immature morphology or a poorly defined core structure (Fig. 6, H44C; ΔZF2). NC mutant H23H44C and ΔZF1ZF2 had often an immature morphology or contained a small core-like structure located close to the viral envelope (Fig. 6, H23H44C and ΔZF1ZF2). The arrows indicate the electron-dense structure at the PM of ΔZF1ZF2-NC mutant, showing an accumulation of mutated Gag unable to complete particle assembly and release.

Data presented in Table 2 show that HIV-1 particles had a canonical morphology with either a conical or a rod-like core with with a mean particle diameter of about 110 nm (Table 2). All the ZF NC mutant particles displayed drastic changes in the core structure with sometimes an immature-like morphology (Fig. 6, see H44C, ΔZF2, H23H44C and ΔZF1ZF2) and displaced or poorly defined cores or even two core structures (Table 2). All mutant virions harbored a defect in the conical shape of the core (Table 2) that can be correlated with the defect in Gag maturation (Fig. 1B, lanes V). In the case of HIV-1 ΔZF1ZF2, the mutant has no distinct core and seemed to accumulate at the budding site but unable to complete the process since we observed electron-dense curvatures of the PM reminiscent of an accumulation of Gag at the PM (Fig. 6, see white arrows).

**Table 2:** Quantitative analysis of virus core morphology of NC mutant HIV-1 particles produced by HeLaP4 cells

	Dense cone shape core	Dense material in base of cone	Round centered core	**Round displaced core**	Tubular core structure	Two core structures or membrane*	No defined core	**Diameter of particles**
wt	**19**		**38**	21	1	0	7	**113 ± 4**
H23C	7	16	16	**40,5**	6	0	14,5	**134 ± 14**
H44C	1	7	5	**45**	13	1	28	**129 ± 14**
H23-H44C	0	0	13	16	23	15*	**33**	**96 ± 12**
ΔZF1	6	16	15	**39**	4	0	20	**131 ± 12**
ΔZF2	1	8	3	**62**	6	2	17	**ND**
ΔZF1-ZF2	1	0	11	23,5	13	22,5*	**29**	**101 ± 13**

Number of particles observed: 80 to 200. Numbers are expressed in % of total observed particles.

Taken together, these results show that HIV-1 NC zinc fingers play an important role in viral core structure and suggest that NC-NC and/or NC-gRNA interactions are essential for HIV-1 Gag assembly and particle release.

## Discussion

The retroviral Gag polyprotein orchestrates retrovirus assembly in the infected cell via two platforms, which are a cellular membrane and a RNA (reviewed in [2,6]. The current view of the assembly process implies that the newly made Gag binds to the cellular membrane by the N-terminal myristoylated domain and stretches of basic residues of the matrix domain (reviewed in [31,32], also involving inositol phosphates/Gag interactions [33-36]. At the same time, the NC domain selectively binds the gRNA via specific interactions with the packaging Psi signal [4], which in turn promote Gag oligomerization [15]. Although, a leucine zipper motif could functionally, at least in part, replace NC to drive the assembly of a "minimal" Gag [37]. We propose that the interactions between Gag-NC and the genomic Psi signal will ensure both the formation of Gag oligomers and the selective recruitment of the gRNA. Consistent with this view, mutations in the first NC zinc finger or the flanking basic residues result in a strong decrease of viral particle production and infectivity [1,16,17,19,38]. Thus, it has been proposed that the NC domain of Gag is required for the proper assembly and release of infectious virions.

To confirm the multiple roles played by NC in HIV-1 assembly, we have examined the role of the NC zinc fingers (ZF) in Gag trafficking. Taken together, our results show that both NC zinc fingers play critical roles in the ability of Gag to properly assemble and ultimately to bud. In fact, all HIV-1 ZF mutants examined so far produced particles at levels five to ten fold, or more, lower than that of wild-type HIV-1 and were not infectious (Table 1).

In model cell lines, the wild-type HIV-1 Gag was found to accumulate either at the PM or on intracellular tetraspanin-rich endosomal membranes as recently reported [8,25,30,39-43]. Mutating the NC Zinc fingers caused Gag to accumulate within the cell, in a diffuse manner and at the PM (Fig. 2). Thus, mutating the NC ZF appears to prevent Gag targeting to and accumulation in endosomes (Fig. 3). This favors the view that endosomes are an important site for virus formation and release (Fig. 3), and also maturation since intracellular mature CAp24 was drastically reduced in the case of the ZF mutants (Fig. 1B). It also indicates that NC is not the major determinant for Gag targeting to the PM, such as the basic MA domain of Gag and phosphatidyl inositol phosphate lipids are [8,30,34,44,45]. However, NC contributes to the localization of Gag in late endosomes (Fig. 3 and 5).

As already stated, the other platform necessary for virus assembly is the gRNA since it directs the oligomerization of the newly made Gag upon binding [15,46,47]. As previously shown for mutations in the first ZF [1], we found that mutating the second ZF and both of them strongly impaired gRNA content in mutant virions as well as particle release (Table 1), and resulted in the production of replication defective viruses. Staufen, a cellular RNA-binding protein involved in RNA transport and metabolism, was reported to interact with both the gRNA and the NC domain of HIV-1 Gag, and to have a role in HIV-1 gRNA encapsidation and Gag assembly [48,49]. Mapping the interaction domain between Staufen and Gag reveals the importance of the NC domain, in particular the second ZF [48,49]. This could explain, at least in part, the role played by the second ZF on gRNA encapsidation if Staufen is responsible for gRNA trafficking to the assembly site and consequently RNA-dependent Gag oligomerization and assembly.

However, Ott and colleagues reported that the decrease in viral production due to NC deletion can be compensated by an RNA binding site in HIV-1 MA domain of Gag [50]. It is very difficult to dissociate the role of NC in virus assembly from NC-RNA interactions, which are critical for the structure of the viral particle [22,51], rather than assembly or budding [16,17]. Our data favor a model in wich all events are linked and dependent upon Gag-RNA interactions via the NC domain, required to achieve a proper viral assembly, i.e. multimerization of Gag, Gag oligomer targeting and trafficking, and ultimately particle assembly, budding and release.

To better examine the role of Gag-RNA interactions in assembly, we examined gRNA localization by FISH (Fig. 4) and by subcellular fractionation (Fig 5). The gRNA was found in the nucleus, most probably due to provirus transcription, in the cytoplasm and at the PM of cells expressing wild-type HIV-1 (Fig. 4), in agreement with Berthold and Mandarelli [52]. Wild-type Gag and the gRNA were also found at the level of late endosomes [25,53,54]. Mutating the NC ZF motifs drastically altered the cellular distribution of the gRNA, because it was found evenly distributed within the cell and no longer associated with the PM (Fig. 4, by FISH) or the late endosomes (Fig. 5, by gradient), while the NC-mutated Gag accumulated mainly at the PM (Fig. 3 and 5). Thus, specific Gag-gRNA interactions via the NC-ZF are most probably required for proper Gag trafficking through Gag-Gag multimer complexes. In agreement with this view, it was reported that Gag expressed from Psi(-) RNA diffuses throughout the cell and shows delayed cytoplasmic colocalisation with the gRNA [54]. The authors propose that the packaging signal may coordinate capture of the genomic Psi(+) RNA by Gag, followed by assembly and transport to the budding site. This data indeed mirrors the results obtained with the NC mutants, in particular the Gag-ΔNC mutant, for which both the gRNA and mutated Gag were found diffuse throughout the cell and had probably lost Gag-gRNA association (as seen in Fig. 4 and 5). Taken together, these data strongly suggest that the specific Gag-gRNA interactions via the NC domain are necessary for proper Gag trafficking and assembly, for Gag oligomers to be targeted to either specific domains at the PM or to the late endosomes.

Finally, the structure of the HIV-1 virions carrying one or several mutations in the NC ZF was determined by electron microscopy (Fig. 6). Results show that the NC mutated virions have lost their conical core shape and are immature, which confirm the role of NC in HIV-1 virion structure [1,16], as well as for other retroviruses, such as SIV [55] and MLV [17]. Our results correlate with previous studies on the assembly of several retroviruses, namely HIV, MLV, RSV that have revealed a critical role of NC in virus assembly and release [1,16,17,21,56,57].

In conclusion, the HIV-1 NC protein appears to be a major actor in the late steps of the virus replication in addition to its roles in proviral DNA synthesis and variability [4,58-60].
